# Free MoS_2_ Nanoflowers Grown on Graphene by Microwave-Assisted Synthesis as Highly Efficient Non-Noble-Metal Electrocatalysts for the Hydrogen Evolution Reaction

**DOI:** 10.1371/journal.pone.0161374

**Published:** 2016-08-24

**Authors:** Jiamu Cao, Xuelin Zhang, Yufeng Zhang, Jing Zhou, Yinuo Chen, Xiaowei Liu

**Affiliations:** 1 MEMS Center, School of Astronautics, Harbin Institute of Technology, Harbin, 150001, P. R. China; 2 School of Electronics and Information Engineering, Harbin Institute of Technology, Harbin, 150001, P. R. China; SPECS Surface Nano Analysis GmbH, GERMANY

## Abstract

Advanced approaches to preparing non-noble-metal electrocatalysts for the hydrogen evolution reaction (HER) are considered to be a significant breakthrough in promoting the exploration of renewable resources. In this work, a hybrid material of MoS_2_ nanoflowers (NFs) on reduced graphene oxide (rGO) was synthesized as a HER catalyst via an environmentally friendly, efficient approach that is also suitable for mass production. Small-sized MoS_2_ NFs with a diameter of ca. 190 nm and an abundance of exposed edges were prepared by a hydrothermal method and were subsequently supported on rGO by microwave-assisted synthesis. The results show that MoS_2_ NFs were distributed uniformly on the remarkably reduced GO and preserved the outstanding original structural features perfectly. Electrochemical tests show that the as-prepared hybrid material exhibited excellent HER activity, with a small Tafel slope of 80 mV/decade and a low overpotential of 170 mV.

## Introduction

Recently, nanosized MoS_2_ has attracted extensive attention as an effective hydrogen evolution reaction (HER) catalyst because of its excellent electrocatalytic properties [1–5]. Computational and experimental studies have confirmed that the HER activity of nanosized MoS_2_ stems from its sulfur edges [6]. As a result, the electroactivity of nanosized MoS_2_ increases with increasing number of exposed edges [7,8]. Different MoS_2_ nanostructures, including nanoparticles (NP), nanosheets (NS) and nanoflowers (NF), have been extensively studied to improve the activity [9,10]. However, the intrinsic poor conductivity of MoS_2_ greatly diminishes its electroactivity by limiting the electron-transfer efficiency. Moreover, the strong van der Waals interactions among lamellar MoS_2_ particles can result in their aggregation, decreasing the number of the exposed edges as well as the electroactivity.

A commonly adopted solution to the aforementioned problems is to fabricate nanosized MoS_2_ on a highly conductive substrate [11]. Graphene nanosheets can act as good substrates because of their large surface area, excellent electrical conductivity and stable chemical properties [12,13]. A hybrid catalyst of MoS_2_ NFs supported on reduced graphene oxide (rGO) shows HER activity with an overpotential of -190 mV and a Tafel slope of 95 mV per decade [14]. To further enhance the conductivity, Cu nanoparticles have been incorporated into MoS_2_/rGO hybrids, which resulted in a decreased Tafel slope of 90 mV per decade [15]. Notably, however, MoS_2_ NFs are still intended to assemble in large quantities on rGO during the fabrication process. Another potential danger is that an inadequate rGO reduction will leave too many oxygen-containing functional groups on the graphene plane, leading to decreased conductivity. Therefore, the development of a hybrid catalyst characterized by a good dispersion of MoS_2_ NFs and sufficient reduction of GO is necessary.

In this study, MoS_2_ NFs prepared by a hydrothermal method were supported on rGO using a microwave-assisted synthesis method with ethylene glycol (EG) as the reducing agent [16,17]. The physical properties of the as-prepared hybrid were characterized by transmission electron microscopy (TEM), energy-dispersive spectrometry (EDS) and X-ray photoelectron spectroscopy (XPS). The HER activity of the hybrid was evaluated by linear sweep voltammetry in 0.5 M H_2_SO_4_ solution at room temperature.

## Experimental

### Materials Preparation

Graphene oxide (GO) was prepared following the Hummer’s method. In the preparation of MoS_2_ NFs, 0.25 mol Na_2_MoO_4_·2H_2_O and 0.98 mol CH_4_N_2_S were dissolved in 30 ml of deionized (DI) water and sonicated to form a clear solution. The precursor solution was transferred into a 50 ml Teflon-lined autoclave, which was subsequently maintained at 185°C for 20 h. The product was collected by centrifugation at 6000 rpm, washed with DI water and ethanol, and vacuum-dried at 60°C.

**Fig 1:**
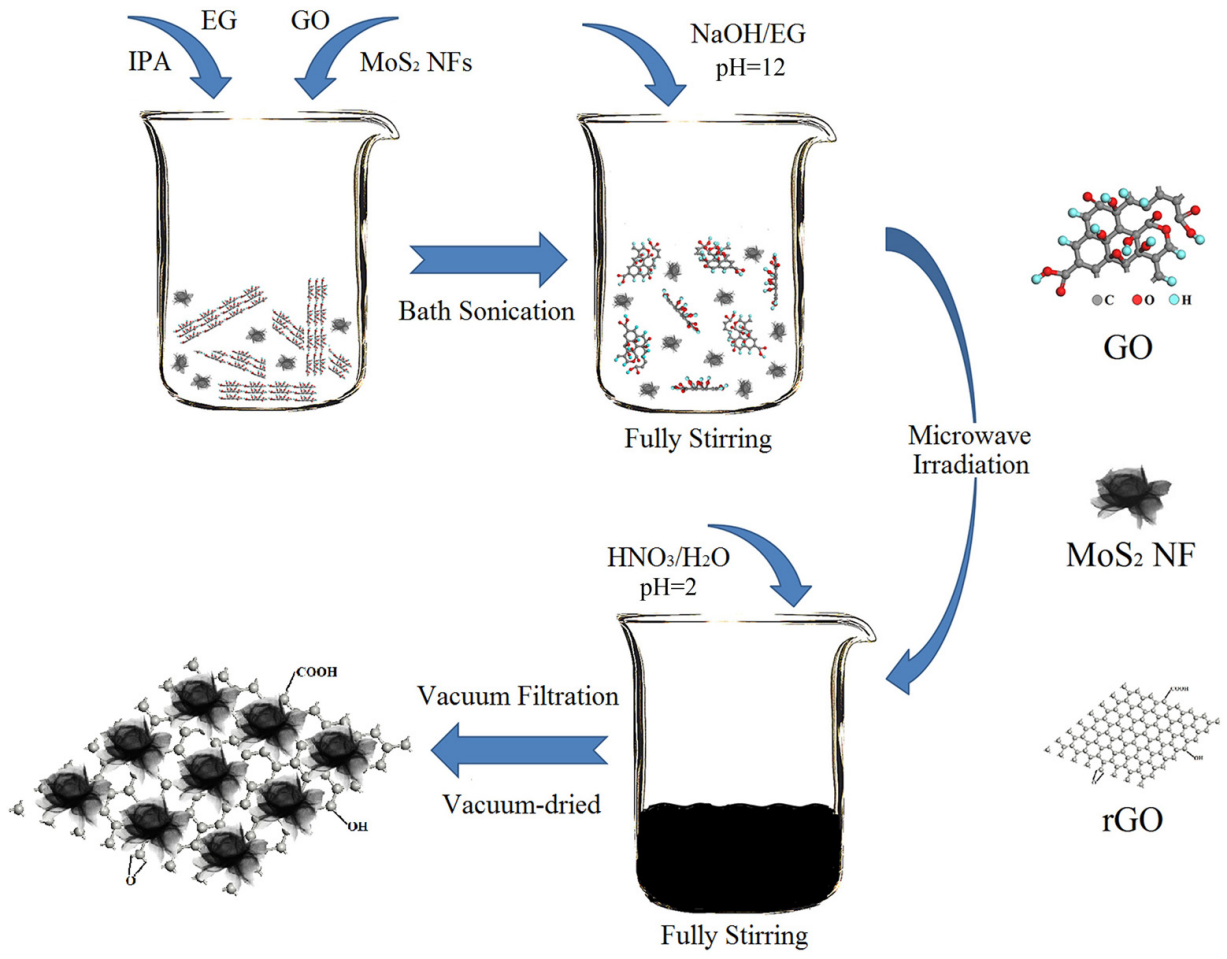
MoS_2_ NFs/rGO hybrid preparation.

The MoS_2_ NFs/rGO hybrid was prepared as follows. As shown in Fig 1, 20 mg GO and 3 mg MoS_2_ NFs were added to 60 ml of a mixture solution of isopropyl alcohol (IPA) and ethylene glycol (v/v = 1:4) and sonicated for 90 min. A 1 M NaOH/EG solution was added to the mixture until a pH of 12 was reached, and then argon was blown into the mixture for 20 min. The mixture was microwaved for ca. 30 s to reach 150°C and was allowed to cool naturally to room temperature. Then, 1 M dilute nitric acid was added until a pH of 2 was reached. The product was collected by vacuum filtration and vacuum-dried at 60°C.

### Preparation of the Working Electrode

The catalyst slurry was prepared by ultrasonically dispersing 4 mg of catalyst and 80 μL of 5 wt% Nafion into 1 ml of 4:1 v/v water/ethanol. A glassy carbon electrode (GCE) with the diameter of 3 mm was polished with alumina suspensions and served as the working electrode. Five microliters of the dispersion were dropped onto the top of the GCE, which was subsequently dried at room temperature for 2 h.

### Structural Characterization and Electrochemical Measurements

The morphologies of the electrocatalysts were observed by TEM using a microscope operated at an accelerating voltage of 300 keV. XPS was used to record the elemental composition and the electron binding energy using a K-Alpha (Thermo Fisher Scientific Company) instrument. The oxygen content was obtained by EDS using a 15 kV electron beam. The HER activity of the MoS_2_ NFs/rGO was examined using linear sweep voltammetry (LSV) with a scan rate of 5 mV s^-1^ in 0.5 M H_2_SO_4_ solution at room temperature. The LSV was carried out using a CHI660D electrochemical workstation in a standard three-electrode setup with a saturated calomel electrode (SCE) as the reference electrode and a Pt foil as the counter electrode. Prior to the electrochemical measurements, the electrolyte was degassed by bubbling of argon gas for 1 h; stable polarization curves were recorded after 20 cycles.

## Results and Discussion

**Fig 2:**
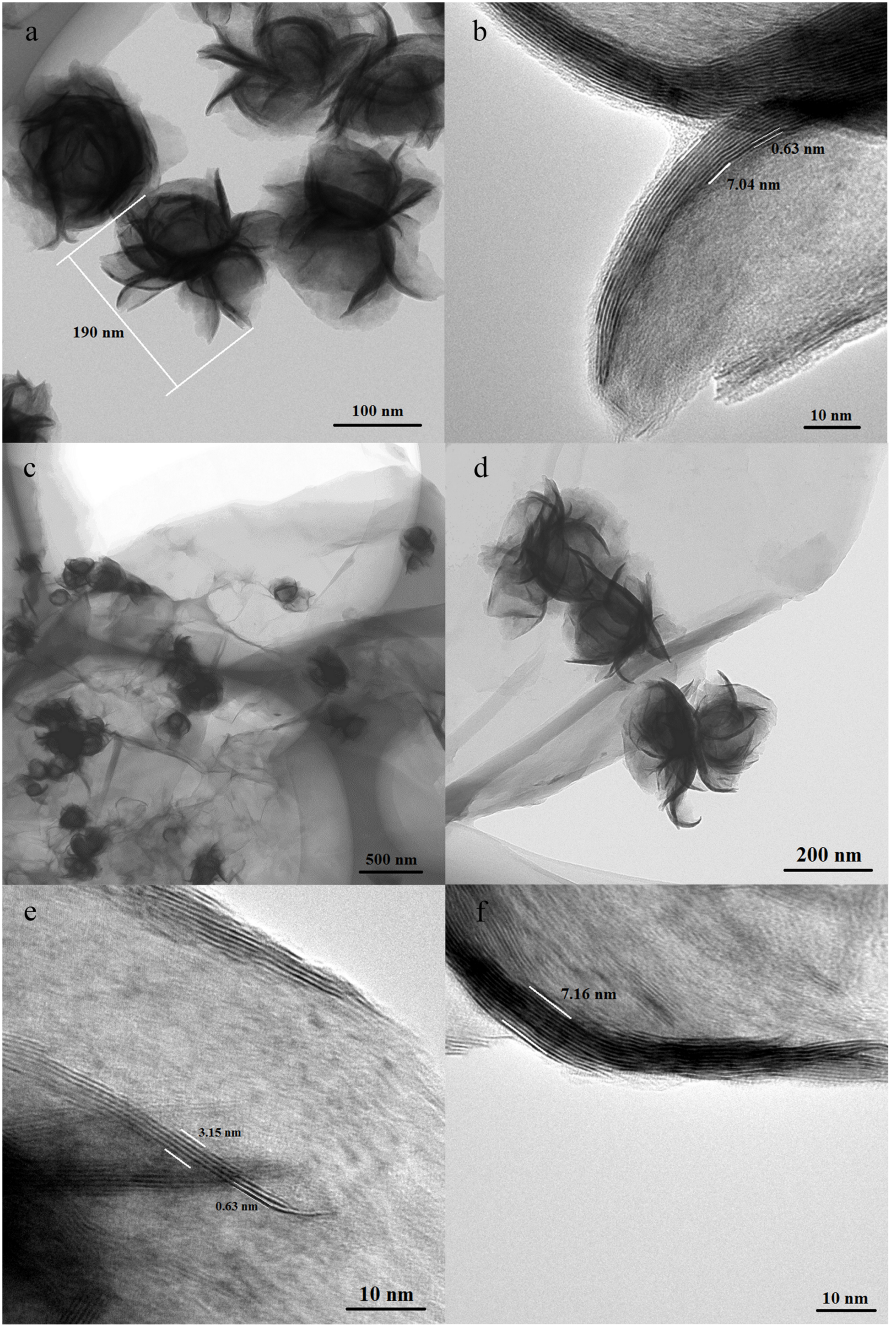
TEM images of MoS_2_ NFs (a), MoS_2_ NFs edges (b), dispersion of MoS_2_ NFs on rGO (c, d) and MoS_2_ NFs edges on rGO (e, f).

The microstructure of MoS_2_ NFs prepared by the hydrothermal method was characterized by TEM, as shown in Figs 2a and 1b. Two distinct characteristics are observed from the two images. First, no obvious aggregation is observed between the flower structures of nanosized MoS_2_; the MoS_2_ exhibits an interlayer spacing of 0.63 nm, and the thickness of MoS_2_ NF petals is 7.04 nm. The links between the MoS_2_ NFs are thin and small, which means that the links between MoS_2_ NFs can be broken under a low energy and a large number of independent MoS_2_ NFs can be easily obtained, thereby providing a solid foundation for the preparation of hybrid materials with good dispersion of MoS_2_ NFs on rGO. Additionally, the size of the prepared MoS_2_ NFs is 190 nm, which is smaller than MoS_2_ NFs previously reported [18]; thus, the as-prepared MoS_2_ NFs likely have more exposed edges than the previously reported materials. Fig 2c shows that rGO sheets reduced by microwave-assisted synthesis exhibit a typical two-dimensional structure and that MoS_2_ NFs are supported on rGO with a uniform distribution. No obvious aggregation of MoS_2_ was observed on the hybrid materials. This result is attributable to the glycolic acid, the product of glycol-reduced rGO, absorbing compactly around the MoS_2_ NFs and preventing their agglomeration. When compared to the NFs in Fig 2a, no obvious change in the size of NFs is observed in Fig 2d. Observation under high magnification revealed that numerous exposed edges could be thick or thin. Thin areas have only 5 MoS_2_ sheets with a thickness of 3.15 nm (Fig 2e), whereas thick areas have 12 MoS_2_ sheets with a thickness of 7.16 nm (Fig 2f). The homogenous and flat layers show that MoS_2_ NFs possess numerous exposed edge sites.

**Fig 3:**
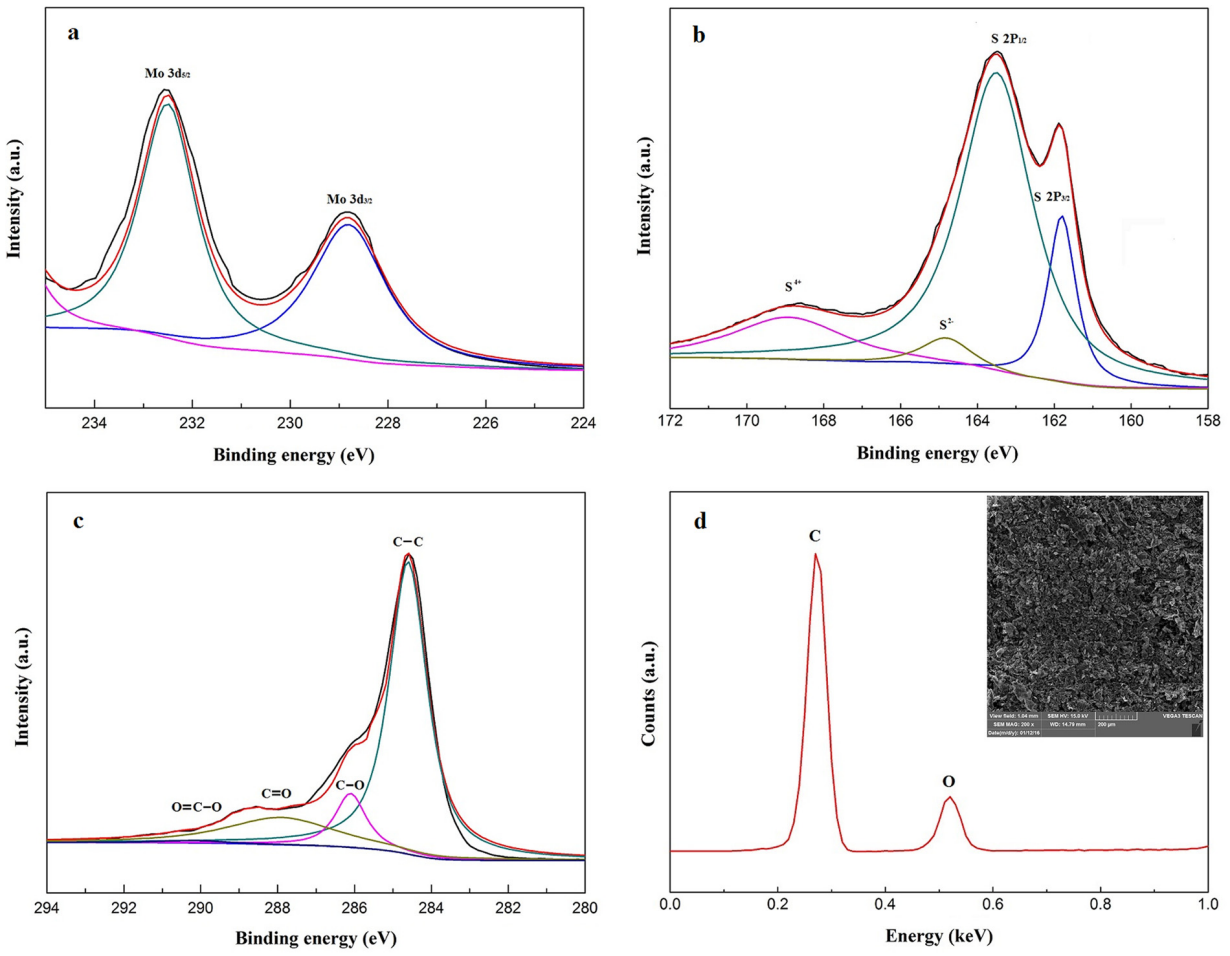
XPS images of Mo 3d (a), S 2p (b), and GO (c). EDS pattern of rGO (d).

XPS was used to investigate the chemical and electronic states of the elements in the material. Fig 3a shows two peaks at Mo^4+^ 3d_5/2_ (228.8 eV) and Mo^4+^ 3d_3/2_ (232.5 eV) binding energies. These peak positions are consistent with the data mentioned in Yan’s report, confirming that the peaks are characteristic of MoS_2_ [19]. Regarding sulfur in MoS_2_, the main peaks at 161.8 eV and 163.5 eV correspond to 2p_3/2_ and 2p_1/2_ binding energies (Fig 3b), and the peak located at 164.8 eV suggests the existence of S^2-^. The intense binding energy at 168.9 eV originates from S^4+^ in sulfate radicals, and these sulfate groups may be exposed at the edge of MoS_2_ layers. The Mo-to-S atomic radio estimated from the XPS spectrum is approximately 1.77.

The two characteristic peaks located approximately at 287.9 eV and 286.1 eV in Fig 3c are assigned to the C = O and C−O−C oxygenated functional groups. A closer observation of Fig 3c reveals a peak at 290.2 eV indicating the presence of the O−C = O functional group; this peak is small but is attributed to the carbon species. The intense peak at 284.6 eV represents the C−C binding energy. The relatively small integrated peak areas of the C = O and C−O−C peaks demonstrate the low oxygen content of the sample. The peak areas of all of these oxygenated functional groups account for less than 30% of the C−C signal. These results indicate that the C/O ratio is approximately 4.09, significantly higher than that of GO.

The C/O ratio was also measured by EDS, as shown in Fig 3d. The average atomic percentages of carbon and oxygen are approximately 80.68% and 19.32%, respectively. The carbon content is three times the oxygen content in the rGO. The C/O ratio is approximately 4.18, as confirmed by the XPS results. The literature contains many reports on graphene oxide that has been well-reduced to graphene. According to Chen’s study, the C/O ratio changed from 2.09 to 5.46 after reduction and the reduced GO exhibited better conductivity, indicating a high degree of reduction [20]. These results confirm that the GO sheets have been efficiently deoxygenated and reduced to graphene.

**Fig 4:**
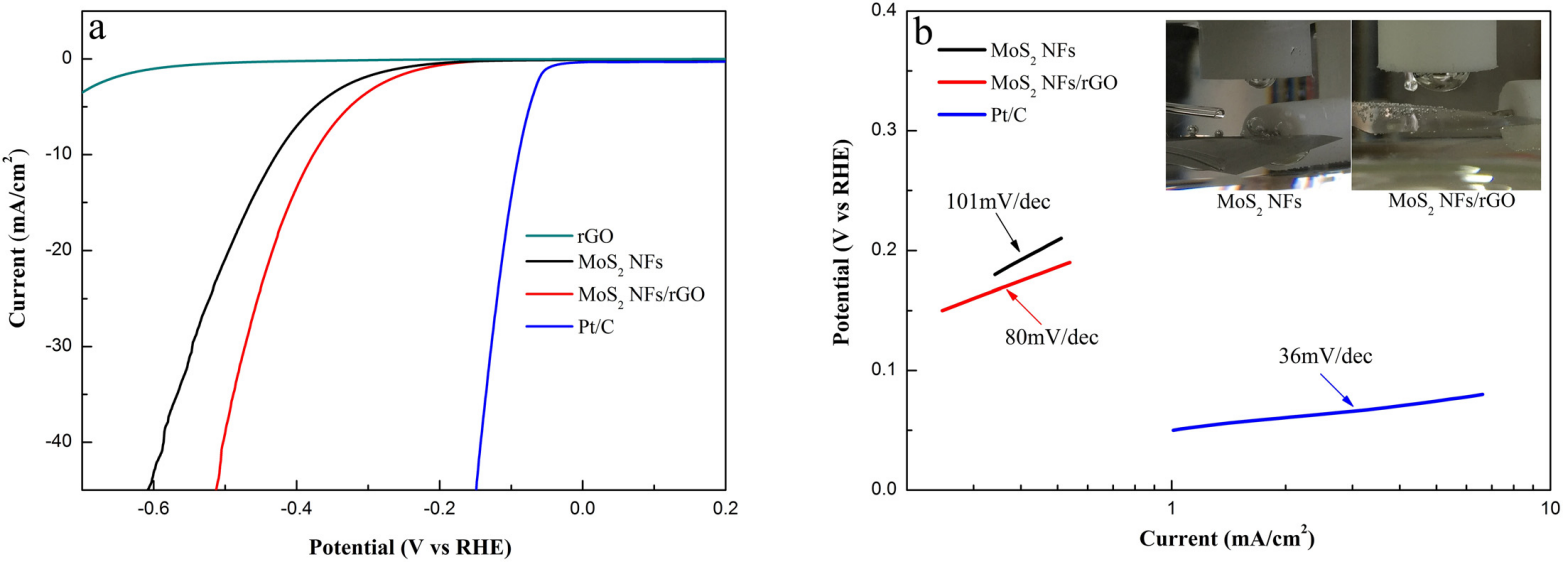
(a) Several catalysts polarization curves and MoS_2_ NFs/rGO hybrid catalyst durability test. (b) Several catalysts Tafel plots (overpotential versus log current). Inset in (b) shows the bubble generated by MoS_2_ NFs and MoS_2_ NFs/rGO in a cycling.

The electrocatalytic HER activities of the MoS_2_ NFs/rGO hybrid material were investigated by polarization curves, as shown in Fig 4a, where a commercial Pt/C catalyst (20 wt% Pt on Vulcan carbon black) is included for comparison. The Pt/C catalyst exhibits high HER catalytic performance, with a near-zero overpotential. The as-prepared MoS_2_ NFs/rGO hybrid gives a low overpotential of 170 mV. In sharp contrast, MoS_2_ NFs or rGO alone exhibited little HER activity.

The linear segments of the Tafel plots (Fig 4b) were fit to the Tafel equation (*η* = *b*∙lg *j* + *a*, where *j* is the current density and *b* is the Tafel slope), yielding Tafel slopes of 101, 80 and 36 mV/decade for MoS_2_ NFs, MoS_2_ NFs/rGO hybrid, and Pt/C, respectively. To get a direct comparison, after a cycling, the bubble generated by MoS_2_ NFs/rGO was significantly larger than that produced by MoS_2_ NFs (Fig 4b). It is further proving that hydrogen evolution reaction performance of MoS_2_ NFs is effectively improved through introducing rGO by microwave assisted synthesis.

The as-prepared hybrid exhibited better properties than those of the hybrids prepared in previous studies [14,15]. This improvement can be ascribed to the following reasons. TEM results reveal that the MoS_2_ NFs supported on rGO are small (190 nm) and uniformly distributed, without aggregation. Such a structure provides more exposed edges and, thus, more active sites for the HER. By contrast, the XPS results clearly show that a higher reduction degree of GO was achieved in the hybrid, resulting in enhanced conductivity of the MoS_2_ NFs/rGO catalyst and minimization of the parasitic Ohmic losses.

## Conclusions

In conclusion, we have reported an improved method for the preparation of MoS_2_ NFs/rGO hybrid, i.e., the preparation of nanosized MoS_2_ NFs via a hydrothermal method and their subsequent deposition on rGO through microwave-assisted synthesis. The hybrid material prepared by this method confers the properties of small size, uniform distribution and large edge positions on the nanosized MoS_2_. The C/O ratio in the prepared MoS_2_ NFs/rGO hybrid is between 4.09 and 4.18, revealing a high degree of reduction for the GO in the hybrid. These structural characteristics result in high HER catalytic activity of the as-prepared MoS_2_ NFs/rGO hybrid, with a low overpotential of 170 mV and a small Tafel slope of 80 mV per decade. We expect the MoS_2_ NFs/rGO hybrid described here to be useful because of its good catalytic properties, excellent electrical conductivity, and good performance as an inexpensive HER electrocatalyst.

## Supporting Information

S1 File Raw data of Fig 4a. (XLS)

S2 File Raw data of Fig 4b. (XLS)
